# *Alpinia oxyphylla* Miq extract reduces cerebral infarction by downregulating JNK-mediated TLR4/T3JAM- and ASK1-related inflammatory signaling in the acute phase of transient focal cerebral ischemia in rats

**DOI:** 10.1186/s13020-021-00495-2

**Published:** 2021-08-21

**Authors:** Chin-Yi Cheng, Su-yin Chiang, Shung-Te Kao, Shang-Chih Huang

**Affiliations:** 1 School of Post-Baccalaureate Chinese Medicine, College of Chinese Medicine, China Medical University, Taichung, 40402 Taiwan; 2 Department of Chinese Medicine, Hui-Sheng Hospital, Taichung, 42056 Taiwan; 3 School of Chinese Medicine, College of Chinese Medicine, China Medical University, Taichung, 40402 Taiwan; 4 Department of Neurology, China Medical University Hospital, Taichung City, 40447 Taiwan

**Keywords:** *Alpinia oxyphylla* Miq, Ischemia, Reperfusion, c-Jun N-terminal kinase, Toll-like receptor 4, TRAF3-interacting JNK-activating modulator, Apoptosis signal-regulating kinase 1

## Abstract

**Background:**

Post-ischemic inflammation is a crucial component in stroke pathology in the early phase of cerebral ischemia–reperfusion (I/R) injury. Inflammation caused by microglia, astrocytes, and necrotic cells, produces pro-inflammatory mediators and exacerbates cerebral I/R injury. This study evaluated the effects of the *Alpinia oxyphylla* Miq [Yi Zhi Ren (YZR)] extract on cerebral infarction at 1 day after 90 min of transient middle cerebral artery occlusion (MCAo) and investigated the molecular mechanisms underlying the regulation of c-Jun N-terminal kinase (JNK)-mediated inflammatory cascades in the penumbral cortex. Rats were intraperitoneally injected with the YZR extract at the doses of 0.2 g/kg (YZR-0.2 g), 0.4 g/kg (YZR-0.4 g), or 0.8 g/kg (YZR-0.8 g) at MCAo onset.

**Results:**

YZR-0.4 g and YZR-0.8 g treatments markedly reduced cerebral infarction, attenuated neurological deficits, and significantly downregulated the expression of phospho-apoptosis signal-regulating kinase 1 (p-ASK1)/ASK1, tumor necrosis factor receptor-associated factor 3 (TRAF3), TRAF3-interacting JNK-activating modulator (T3JAM), ionized calcium-binding adapter molecule 1 (Iba1), p-JNK/JNK, inducible nitric oxide synthase, cyclooxygenase-2, tumor necrosis factor-α, toll-like receptor 4 (TLR4), glial fibrillary acidic protein (GFAP), nuclear factor-kappa B (NF-κB), and interleukin-6 in the penumbral cortex at 1 day after reperfusion. SP600125 (SP), a selective JNK inhibitor, had the same effects. Furthermore, Iba1- and GFAP-positive cells were colocalized with TLR4, and colocalization of GFAP-positive cells was found with NF-κB in the nuclei.

**Conclusion:**

YZR-0.4 g and YZR-0.8 g treatments exerted beneficial effects on cerebral ischemic injury by downregulating JNK-mediated signaling in the peri-infarct cortex. Moreover, the anti-infarction effects of YZR extract treatments were partially attributed to the downregulation of JNK-mediated TLR4/T3JAM- and ASK1-related inflammatory signaling pathways in the penumbral cortex at 1 day after reperfusion.

## Background

Post-ischemic inflammation is a crucial component in stroke pathology in the early phase of cerebral ischemia–reperfusion (I/R) injury [1]. The increased production of free radicals and the consequent induction of oxidative stress in the ischemic area initiate inflammatory responses [2]. Generally, inflammation is caused by microglia, astrocytes, and necrotic cells, producing pro-inflammatory mediators and aggravating cerebral I/R injury [3]. In the pathological process of cerebral ischemia, toll-like receptor (TLR)-mediated signaling initiates inflammatory cascades and is closely related to the development of cerebral infarction [4].

TLRs play an important role in the innate immune response. TLR4, a member of the TLR family, is expressed on microglia and astrocytes in the ischemic area in cerebral I/R injury [5]. During brain ischemic insult, activated microglia and reactive astrocytes predominantly express TLR4, which recognizes damage-associated molecular patterns (DAMPs), subsequently triggering downstream cascades through myeloid differentiation primary response gene 88 (MyD88)-dependent and toll/interleukin (IL)-1 receptor homology domain-containing adaptor-inducing interferon-β (TRIF)-dependent signaling pathways [6, 7]. These two pathways result in the activation of transcription factor nuclear factor-kappa B (NF-κB), which stimulates the production of pro-inflammatory mediators [6, 8]. The expression of TLR4, ionized calcium-binding adapter molecule 1 (Iba1; a marker of microglia), and glial fibrillary acidic protein (GFAP; a marker of astrocytes) is markedly increased in the ischemic area at 24 h after transient focal cerebral ischemia [9]. In MyD88-dependent signaling, MyD88 interacts with tumor necrosis factor (TNF) receptor-associated factor 6 (TRAF6), subsequently activating two pathways involving NF-κB- and mitogen-activated protein kinase (MAPK), which includes extracellular signal-regulated kinase 1/2, c-Jun N-terminal kinase (JNK), and p38 MAPK [10, 11]. In TRIF-dependent signaling, TRIF binds to TRIF-related adaptor molecule (TRAM), subsequently activating TRAF3 and resulting in the activation of interferon-β expression [12, 13]. Studies have reported that TRAF3-interacting JNK-activating modulator (T3JAM) (also named TRAF3IP3), a coiled-coil membrane protein, interacts with TRAF3 in the cytosol, subsequently triggering the activation of JNK-mediated signaling and further amplifying TLR4-mediated signaling [14, 15]. In the early phase of cerebral ischemia, oxidative stress triggers the activation of apoptosis signal-regulating kinase 1 (ASK1), which activates downstream MAPK kinase (MKK) 4/JNK signaling cascades, leading to the initiation of apoptosis and inflammation in the ischemic area [16, 17]. JNK signaling plays a crucial role in modulation of multiple cellular activities, including proliferation, differentiation, inflammation, and apoptosis [18]. Furthermore, JNK is considered a major stress-activated protein kinase and a promising candidate for activating microglia, and it induces neuroinflammation in response to I/R injury in in vitro and in vivo models [19].

Generally, JNK pathways are activated by TLR4/MyD88/TRAF6-, TLR4/T3JAM-, and ASK1-mediated signaling stimuli during cerebral ischemia. Phosphorylated JNK positively regulates activated NF-κB, which translocates into the nucleus and induces the expression of genes encoding pro-inflammatory cytokines such as inducible nitric oxide synthase (iNOS), cyclooxygenase-2 (COX-2), TNF-α, and IL-6 [18, 20]. In post-ischemic inflammatory cascades, NF-κB-mediated iNOS production induces oxidative stress and then disrupts blood–brain barrier (BBB) integrity, aggravating cerebral infarction [21]. COX exists in two isoforms: COX-1 and COX-2. COX-1 is a constitutive enzyme and is expressed in most tissues, where COX-1-derived prostanoids provide stability to the internal environment. By contrast, COX-2 is the inducible isoform; it is highly expressed in the cerebral ischemic area and consequently promotes microglia activation, thereby enlarging the cerebral infarct area [22, 23]. Elevated TNF-α expression causes increased disruption of the BBB integrity and stimulates cytotoxic iNOS production by microglia and astrocytes. In addition, TNF-α, in turn, could induce the production of NF-κB, leading to the augmentation of the inflammatory response and exacerbation of brain injury [12, 21, 24]. IL-6 that is mainly produced by activated microglia contributes to BBB disruption and is closely associated with neuronal damage in the ischemia penumbra during transient middle cerebral artery occlusion (MCAo). Furthermore, IL-6 shows peak expression in the ischemic penumbra at 24 h after MCAo [21, 25].

*Alpinia oxyphylla* Miq, commonly known as Yi Zhi Ren (YZR), is a traditional Chinese herb that has been widely used to treat intestinal disorders, urosis, diuresis, ulceration, hypertension, dementia, and cerebrovascular disorders [26, 27]. Studies have reported that YZR attenuates memory impairment through the inhibition of neuroinflammation, amyloid-β deposition, and p-tau expression in the cortex and hippocampus in mice with lipopolysaccharide-induced Alzheimer’s disease [28]. In addition, YZR protects against ischemia-induced memory deficits by promoting hippocampal cornu ammonis neuronal survival after transient global cerebral ischemia [29]. Protocatechuic acid, chrysin and nootkatone are the main active components of YZR [30, 31]. Intraperitoneal (IP) administration of protocatechuic acid (5 mg/kg) contributes to the suppression of oxidative stress by increasing superoxide dismutase and glutathione peroxidase activities and decreasing malondialdehyde expression in the brain in aged rats [32]. Chrysin attenuates cerebral I/R injury by downregulating IL-6, TNF-α, NK-κB, COX-2, and iNOS expression in the ischemic area after transient focal [33] and global [34] cerebral ischemia. Intracerebroventricular (ICV) injections of nootkatone (0.02 mg/kg and 0.2 mg/kg) provide beneficial effects against β-amyloid-induced cognitive impairment by upregulating anti-oxidative and anti-acetylcholinesterase activities in the hippocampus [35]. On the basis of the aforementioned findings, we speculate that the YZR extract protects against cerebral I/R injury by modulating JNK-mediated signaling in the acute phase of transient focal cerebral ischemia. Therefore, in this study the effects of the YZR extract on cerebral infarction were assessed, and the potential mechanism through which the extract modulates JNK-mediated inflammatory signaling in the penumbral cortex at 1 day after transient MCAo was verified.

## Methods

### Experimental animals

A total of 151 adult male Sprague–Dawley rats, 8–9 weeks old and weighting 290–330 g (purchased from BioLASCO Co., Ltd., Yilan, Taiwan), were used in the present study. They were housed under the conditions of controlled temperature (22–24 °C), humidity (50–55%), and lighting cycle (12/12-h light/dark). All experimental procedures were conducted in accordance with the guidelines approved by the Institutional Animal Care and Use Committee of China Medical University (Permit Number: CMUIACUC-2019-312). Twenty three rats died during the experiments and 7 rats subjected to incomplete MCAo were excluded from this study.

### YZR extract preparation

YZR extract powder was obtained from Chuang Song Zong Pharmaceutical Co., Ltd. (Kaohsiung, Taiwan). Two grams of YZR extract powder was dissolved with 8 mL of double-distilled water. The concentration measurements were performed as described previously [36]. The final concentration of the YZR aqueous extract was maintained at 0.1 g/mL.

### High-performance liquid chromatography assessment of the indicators of the YZR extract

The standards comprising protocatechuic acid [purity: 99.9%, National Institutes for Food and Drug Control (NIFDC), China], chrysin (purity: 100%, NIFDC, China) and nootkatone (purity: 99.5%, NATURE STANDARD, Shanghai, China) were precisely weighed and dissolved in absolute methanol to prepare standard solutions. Two grams of the YZR extract powder was dissolved in 100 mL of absolute methanol and the solution was then shaken using an ultrasonic cleaner at room temperature (RT) for 30 min. After filtration of the solution, the filtrate was collected as the sample solution. Subsequently, high-performance liquid chromatography (HPLC) measurements were conducted as described previously [37]. In brief, 20 μL of the standard or sample solution was injected into the Waters HPLC system (Waters Corp., Miford, MA, USA), which consists of the Waters 2690 Separations Module and Waters 2996 Photodiode Array Detector. The HPLC profile of the YZR extract was determined using a C18 column (Cosmosil 5C18-AR-II, 4.6 mm I.D. × 250 mm, 5 μm). The mobile phase consisted of water with 0.1% phosphoric acid (A) and acetonitrile with 0.1% phosphoric acid (B). In gradient elution processes, the proportion of mobile phase A was decreased from 93 to 30%, whereas the proportion of mobile phase B was increased from 7 to 70%. The flow-rate of the mobile phase was 1.0 mL/min, and the total run time was 85 min. The effluent was monitored by a photodiode array detector at 254 nm.

### Transient middle cerebral artery occlusion

Transient MCAo was performed in the rats by using the intraluminal suture occlusion technique described previously [37]. In Brief, all rats were anesthetized with isoflurane (5% and 2% isoflurane for induction and maintenance, respectively). The rat’s head was fixed in the stereotaxic frame and a burr hold was drilled into the skull (2.0 mm posterior and 2.5 mm lateral to the right from the bregma) to expose the distal territory of the middle cerebral artery (MCA). A 3-cm midline neck incision was made to expose the right external carotid artery (ECA) and internal carotid artery (ICA). A 3–0 nylon suture with a heat-blunted tip was carefully inserted into the lumen of the right ICA through the stump of the ECA and was advanced up to the origin of the MCA. After 90 min of MCAo, the suture was gently withdrawn to permit reperfusion. Blood flow in the MCA was monitored using a Laser-Doppler flowmetry (DRT4, Moor Instruments Inc., Wilmington, USA) in the MCAo procedure. Successful establishment of MCAo was defined as a reduction in the MCA blood flow to 20–30% of baseline in the ischemic period and an increase in MCA blood flow to 60% of baseline in the reperfusion period. The rats subjected to incomplete MCAo were excluded for further study.

### Assessment of neurological function

Modified neurological severity score (mNSS) tests were performed to determine neurological function at 1 day after reperfusion. The mNSS tests listed in Table 1 are divided into four components: motor, sensory, beam balance, and reflex tests, as described previously [38]. The neurological deficit scores (NDSs) for each rat were obtained using the mNSS, which ranges from 0 to 18. A normal score is 0, and the maximal deficit score is 18.

**Table 1:** The components of the mNSS

Tests	Scores
Motor tests	
Raising rat by tail	
Forelimb flexion	1
Hindlimb flexion	1
Head moves more than 10 degree from the vertical axis	1
Placing rat on floor	
Walking in a straight line	0
Walking toward the paretic side	1
Circling toward the paretic side	2
Falls down on the contralateral or ipsilateral side	3
Sensory tests	
Placing test	
No forelimb placing response after vibrissae stimulation	1
Proprioceptive test	
No forelimb resistance after pushing paws against the table edge	1
Beam balance tests	
Normal walking on the beam	0
Grasps side of the beam	1
Hugs the beam and the paretic forelimb falls down from the beam	2
Hugs the beam and the paretic forelimb and hindlimb fall down from the beam	3
Attempts to balance on the beam but falls off (more than 40 s)	4
Attempts to balance on the beam but falls off (the duration between 20 and 40 s)	5
Falls off immediately	6
Reflex absence and abnormal movement	
Lack of pinna reflex (examined using a cotton swab into the ear canal)	1
Lack of corneal reflex (examined using a cotton swab lightly touching the cornea)	1
Lack of startle reflex (examined using a loud hand clap)	1
Seizure, myoclonus, or myodystony	1

## Experiment A

### Grouping

The rats were randomly divided into five groups (n = 5–6): Sham, Control, YZR-0.2 g, YZR-0.4 g, and YZR-0.8 g groups. The rats in the YZR-0.2 g, YZR-0.4 g, and YZR-0.8 g groups were IP injected with the YZR extract at the doses of 0.2, 0.4, and 0.8 g/kg, respectively, after the initiation of MCAo. After 90 min of ischemia followed by 1 day of reperfusion, the rats were euthanized by CO_2_ inhalation, and their brains were immediately removed. The rats in the Control group were subjected to the identical protocols of the YZR-0.8 g group, except that the rats were injected with normal saline instead of the YZR extract. The rats in the Sham group were subjected to the identical protocols of the Control group, except that the MCA was not occluded.

### Measurement of cerebral infarction

After 1 day of reperfusion, the rats were euthanized, and their brains were immediately removed. The fresh brains were placed at − 20 °C for 5 min and were then cut into six coronal sections of 2-mm thickness. The brain sections were stained with 2% 2,3,5-triphenyltetrazolium chloride (TTC; Merck, Germany) at 37 °C for 5 min and consequently fixed in 4% paraformaldehyde (PFA) solution at RT overnight. In each TTC-stained brain section, the white portion in the ipsilateral hemisphere indicates the infarcted area, whereas the deep red portion indicates the healthy region. The percentage of cerebral infarct areas was determined by dividing the infarct area by the total coronal sectional area; the calculation was conducted using ImageJ software (NIH, MD, USA).

## Experiment B

### Grouping

The rats were randomly divided into five groups (n = 5): Sham, Control, YZR-0.2 g, YZR-0.4 g, and YZR-0.8 g groups. The rats in these groups were subjected to protocols identical to those for Experiment A.

### Western blot analysis

After 1 day of reperfusion, the rats were euthanized, and their brains were immediately removed. The right penumbral cortices of the brain samples were divided into cytosolic and mitochondrial fractions, as described previously [37]. Equal amounts of protein samples (15 μg/lane) were loaded and then separated through 10% sodium dodecyl sulfate–polyacrylamide gel electrophoresis, transferred onto nitrocellulose (NC) membranes, and incubated with primary antibodies listed in Table 2 at 4 °C overnight. Subsequently, appropriate secondary antibodies (1:5000 dilution) (Table 2) were used to detect the primary antibodies presented in the NC membranes, with incubation at RT for 1 h. The images were scanned using a luminescence image analyzer (LAS-3000, FujiFilm), and the data were analyzed using ImageJ software.

**Table 2:** Primary and secondary antibodies applied in this study

Source	Primary antibody Secondary antibody	WB Dilution	IHC Dilution	IF Dilution	Supplier/product number
Rabbit Goat	p-ASK1 Anti-rabbit IgG	1:1000 1:5000			CST/#3765 Jackson/AB_2313567
Rabbit Goat	ASK1 Anti-rabbit IgG	1:1000 1:5000			CST/#8662 Jackson/AB_2313567
Rabbit Goat	MyD88 Anti-rabbit IgG	1:1000 1:5000			CST/#4283 Jackson/AB_2313567
Rabbit Goat	TRAF6 Anti-rabbit IgG	1:1000 1:5000			abcam/ab40675 Jackson/AB_2313567
Rabbit Goat	T3JAM Anti-rabbit IgG	1:500 1:5000			Merck/SAB4503206 Jackson/AB_2313567
Rabbit Goat	TRAF3 Anti-rabbit IgG	1:500 1:5000			abcam/36988 Jackson/AB_2313567
Rabbit Goat	Iba1 Anti-rabbit IgG	1:1000 1:5000			abcam/ab178846 Jackson/AB_2313567
Rabbit Goat	p-JNK Anti-rabbit IgG	1:1000 1:5000			CST/#9251 Jackson/AB_2313567
Rabbit Goat	JNK Anti-rabbit IgG	1:1000 1:5000			CST/#9252 Jackson/AB_2313567
Rabbit Goat	p-p38 MAPK Anti-rabbit IgG	1:1000 1:5000			CST/#9211 Jackson/AB_2313567
Rabbit Goat	P38 MAPK Anti-rabbit IgG	1:1000 1:5000			CST/#9212 Jackson/AB_2313567
Rabbit Goat	iNOS Anti-rabbit IgG	1:250 1:5000			abcam/ab15323 Jackson/AB_2313567
Rabbit Goat	COX-2 Anti-rabbit IgG	1:1000 1:5000			CST/#4842 Jackson/AB_2313567
Rabbit Goat	TNF-α Anti-rabbit IgG	1:1000 1:5000			Millipore/AB1837P Jackson/AB_2313567
Mouse Goat	Actin (loading control) Anti-mouse IgG	1:5000 1:5000			NOVUS/NB600-501 Jackson/AB_10015289
Rabbit	Iba1 Polymer kit		1:500		abcam/ab178846 Leica/RE7111&RE7112
Mouse	TLR4 Polymer kit		1:100		abcam/ab22048 Leica/RE7111&RE7112
Mouse	GFAP Polymer kit		1:200		CST/#3670 Leica/RE7111&RE7112
Rabbit	NF-κB (p65) Polymer kit		1:200		abcam/ab16502 Leica/RE7111&RE7112
Rabbit	iNOS Polymer kit		1:100		abcam/ab15323 Leica/RE7111&RE7112
Mouse	IL-6 Polymer kit		1:500		abcam/ab9324 Leica/RE7111&RE7112
Mouse Goat	NeuN Anti-mouse IgG			1:100 1:100	abcam/ab178846 Jackson Alexa 594/AB_2338871
Rabbit Goat	Iba1 Anti-rabbit IgG			1:250 1:100	abcam/ab178846 Jackson Alexa 488/AB_2338046
Mouse Goat	TLR4 Anti-mouse IgG			1:100 1:100	abcam/ab22048 Jackson Alexa 594/AB_2338871
Mouse Goat	GFAP Anti-mouse IgG			1:100 1:100	CST/#3670 Jackson Alexa 594/AB_2338871
Rabbit Goat	NF-κB (p65) Anti-rabbit IgG			1:100 1:100	abcam/ab16502 Jackson Alexa 488/AB_2338046
Rabbit Goat	GFAP Anti-rabbit IgG			1:100 1:100	abcam/ab104139 Jackson Alexa 488/AB_2338046

WB: Western blotting; IHC: immunohistochemistry; IF: immunofluorescence; CST: Cell Signaling Technology

## Experiment C

### Grouping

The rats were randomly divided into four groups (n = 5): D+Sham, D+Control, D+YZR-0.8 g, and SP groups. The rats in the SP group were subjected to the Control group protocols identical to those for Experiment A, but they were given an ICV injection of SP600125, a selective JNK inhibitor, 20 min before MCAo. The rats in the D+Sham, D+Control, and D+YZR-0.8 g groups were subjected to protocols identical to those for the Sham, Control, and YZR-0.8 g groups, as described in Experiment A, respectively; however, all of them were given ICV injections of 1% dimethyl sulfoxide (DMSO) 20 min before MCAo.

### Intracerebroventricular injection of SP600125 or 1% DMSO

The rats were maintained under anesthesia with 2% isoflurane, and a burr hole located 0.8 mm posterior to the bregma and 1.5 mm lateral to the midline was drilled into the right side of the skull. The rats were given an ICV injection of 10 μL of SP600125 solution (2 mM in DMSO, ab120065 abcam) or 1% DMSO solution. The solution was injected at a depth of 3.5 mm from the skull using a 10-µL Hamilton syringe (Hamilton Company, Reno, NV, USA).

### Measurement of neurological function and cerebral infarction

One day after reperfusion, the rats were subjected to mNSS tests and were subsequently euthanized for measurement of cerebral infarction. The cerebral infarction measurement procedures are the same as those in Experiment A.

## Experiment D

### Grouping

The rats were randomly divided into four groups (n = 5): D+Sham, D+Control, D+YZR-0.8 g, and SP groups. The rats in these groups were subjected to protocols identical to those for Experiment C.

### Western blot analysis

One day after transient MCAo, the rats were euthanized, and their brains were immediately removed for Western blot analysis of p-ASK1, ASK1, Iba1, T3JAM, TRAF3, p-JNK, JNK, iNOS, COX-2, and TNF-α (listed in Table 2) expression. The procedures of Western blot assay were the same as those for Experiment B.

## Experiment E

### Grouping

The rats were randomly divided into six groups (n = 5): Sham, Control, YZR-0.2 g, YZR-0.4 g, YZR-0.8 g, and SP groups. The rats in these groups were subjected to protocols identical to those for Experiment A and C.

### Immunohistochemical analysis

After completing neurological examinations at 1 day of reperfusion, the rats were euthanized. They were transcardially perfused with cold 0.9% saline, and their brains were removed quickly. Subsequently, the brains were embedded in optimal cutting temperature compound in small aluminum foil paper boxes, frozen at − 35 ± 5 °C using dry ice, and cut into 15 µm thick coronal brain sections using a cryostat (Leica CM3050 S, Wetzlar, Germany), as previously described [36]. The brain sections were rinsed in phosphate buffered saline/Tween 20 (0.01%; PBST) and post-fixed with 4% PFA at RT for 15 min. After washing with PBST, the brain sections were immersed with 3% hydrogen peroxidase/methanol for 20 min to inhibit endogenous peroxidase activity and then incubated with Iba1, GFAP, TLR4, NF-κB, iNOS, and IL-6 (listed in Table 2) at 4 °C overnight. The brain sections were subsequently stained with appropriate secondary antibodies (Table 2) and labeled with avidin–biotin–peroxidase complexes (Leica Biosystems Newcastle Ltd., UK). Immunopositive cells in the selected penumbral cortex were detected using a light microscope (Axioskop 40, Zeiss). The negative control slides from the Control group were stained without the primary antibodies.

### Immunofluorescence staining

The brain sections adjacent to those used in immunohistochemical (IHC) analysis were used for immunofluorescence (IF) staining. The brain sections were rinsed in PBST and post-fixed with 4% PFA at RT for 15 min. After washing with PBST, the brain sections were blocked with a blocking buffer containing 1% bovine serum albumin in PBST at RT for 1 h, and the sections were subsequently incubated with mouse and rabbit primary antibodies (listed in Table 2) at 4 °C overnight. After being washed with PBST for 5 min, the brain sections were incubated with anti-mouse and anti-rabbit immunoglobulin G secondary antibodies (Table 2) at 37 °C for 1.5 h and then counterstained with 4',6-diamidino-2-phenylindole (DAPI, ab4139 abcam) at RT for 3 min, as described previously [36]. Immunopositive cells in the selected penumbral cortex were evaluated using a fluorescence microscope (CKX53, Olympus, Tokyo, Japan). In addition, the percentage of activated forms of Iba1 (GFAP) was evaluated by dividing activated forms of Iba1 (GFAP)-positive cells by total Iba1 (GFAP)-positive cells in the selected penumbral cortex.

### Statistical analysis

The normality test was performed on all data by Kolmogorov–Smirnov test with a significance level of 0.05. All numeric data, except for neurological function scores, follow the normal distribution (*P* > 0.05). The data acquired from cerebral infarction, Western blot, IHC, and IF analyses among the experimental groups were evaluated using one-way analysis of variance (ANOVA) followed by Bonferroni post-hoc test, and the data were expressed as mean ± standard deviation. The data acquired from neurological tests among the experimental groups were analyzed using Kruskal–Wallis one-way ANOVA, and the data were expressed as median (min−max). *P* values less than 0.05 were considered as statistically significant.

## Results

### HPLC analysis of the YZR extract

**Fig.1:**
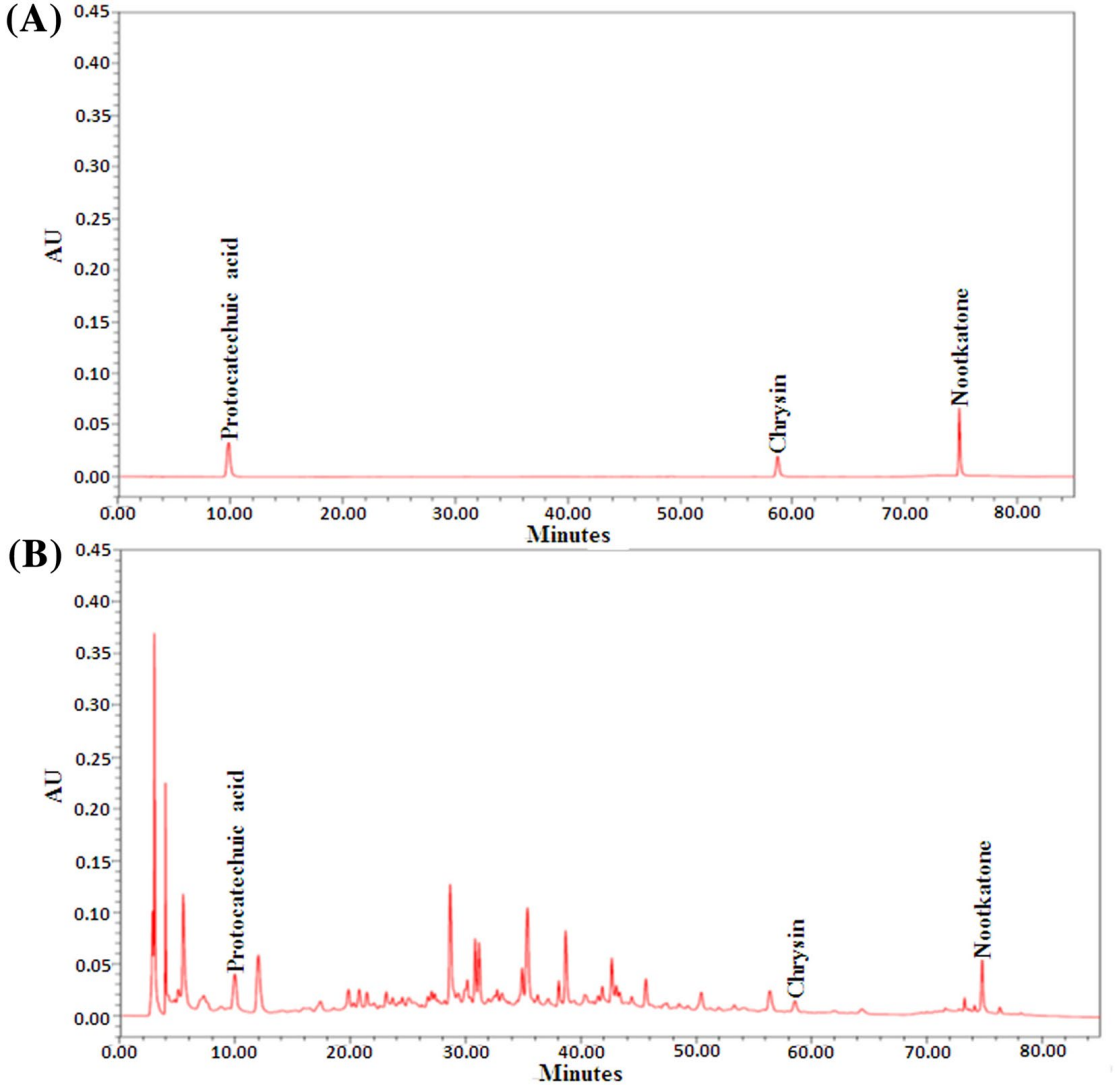
HPLC chromatograms of protocatechuic acid, chrysin, and nootkatone in the YZR extract. HPLC chromatograms **A** and **B** denote the standard and YZR extract solutions, respectively. AU: absorbance unit

After the detection of the standard and YZR extract solutions using HPLC analysis, the retention times of protocatechuic acid, chrysin, and nootkatone were 10.0, 58.7, and 75.0 min, respectively. The contents of protocatechuic acid, chrysin, and nootkatone in the YZR extract were 0.064, 0.017, and 0.083 mg/g, respectively (Fig. 1A and B).

### Effects of YZR extract treatments on cerebral infarction

**Fig. 2:**
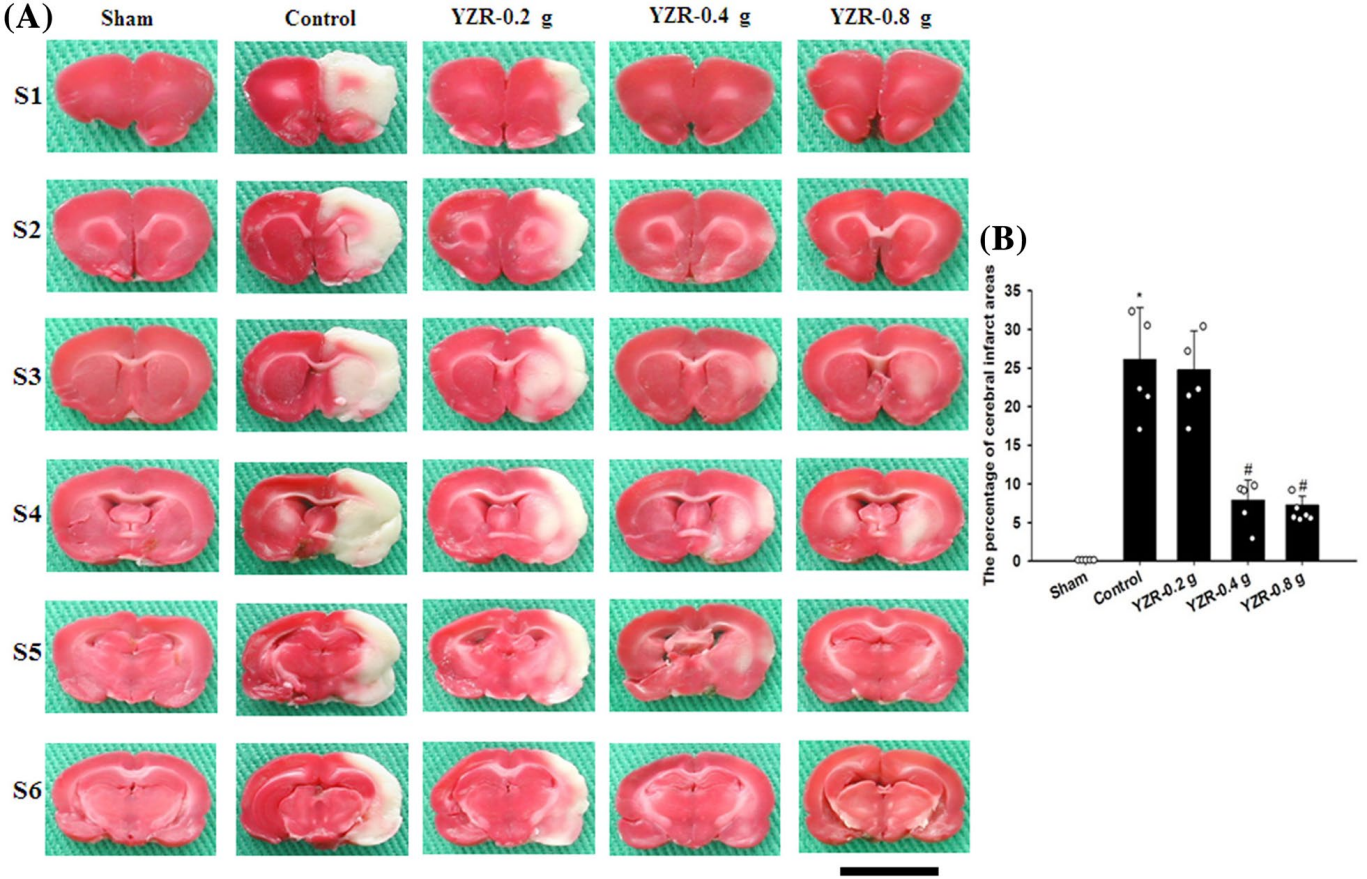
Effects of YZR-0.4 g and YZR-0.8 g treatments on cerebral infarction at 1 day after reperfusion. **A** Representative TTC staining images (S1–S6) of the Sham, Control, YZR-0.2 g, YZR-0.4 g, and YZR-0.8 g groups (n = 5–6) showed that normal tissues turned deep red, whereas infarcted tissues remained pale white. **B** The percentage of cerebral infarct areas was measured among the experimental groups at 1 day after reperfusion. Data are expressed as mean ± standard deviation. *P < 0.05 vs. the Sham group; ^#^*P* < 0.05 vs. the Control group. Scale bar represents 1 cm

After 1 day of reperfusion, TTC staining revealed that the percentage of cerebral infarct areas was markedly higher in the Control group than in the Sham group (*P* < 0.05) and was markedly lower in the YZR-0.4 g and YZR-0.8 g groups than in the Control group (both *P* < 0.05; Fig. 2A and B) (*F*_4,21_ = 45.700, *P* = 0.000). The percentage of cerebral infarct areas did not differ significantly between the Control and YZR-0.2 g groups (*P* > 0.05).

### Effects of YZR extract treatments on neurological function

The mNSS tests revealed that the NDSs of motor, sensory, and beam balance functions were markedly higher in the Control group than in the Sham group (all *P* < 0.05) and were markedly lower in the YZR-0.4 g and YZR-0.8 g groups than in the Control groups (all *P* < 0.05; Table 3). However, the NDSs of motor, sensory, and beam balance functions did not differ significantly between the Control and YZR-0.2 g groups (*P* > 0.05). In addition, the rats in the experimental groups did not lose the reflex function at 1 day after reperfusion.

**Table 3:** The NDSs of neurological tests performed in Experiment A, B, and E (n = 15)

Group	NDSs of motor function	NDSs of sensory function	NDSs of beam balance function
Sham	0 (0–0)	0 (0–0)	0 (0−0)
Control	4 (3–4)*	2 (1–2)*	2 (2−3)*
YZR-0.2 g	4 (3–4)	2 (1–2)	2 (2−3)
YZR-0.4 g	2 (1–3)^#^	1 (0–2)^#^	1 (0−2)^#^
YZR-0.8 g	2 (1–3)^#^	0 (0–1)^#^	1 (0−1)^#^

Each value was expressed as median (min−max)

NDSs: neurological deficit scores

*P < 0.05 vs. the Sham group; ^#^*P* < 0.05 vs. the Control group

### Effects of YZR extract treatments on the cytosolic expression of p-ASK1, ASK1, MyD88, TRAF6, T3JAM, TRAF3, Iba1, p-JNK, JNK, p-p38 MAPK, p38 MAPK, iNOS, COX-2, and TNF-α

**Fig. 3:**
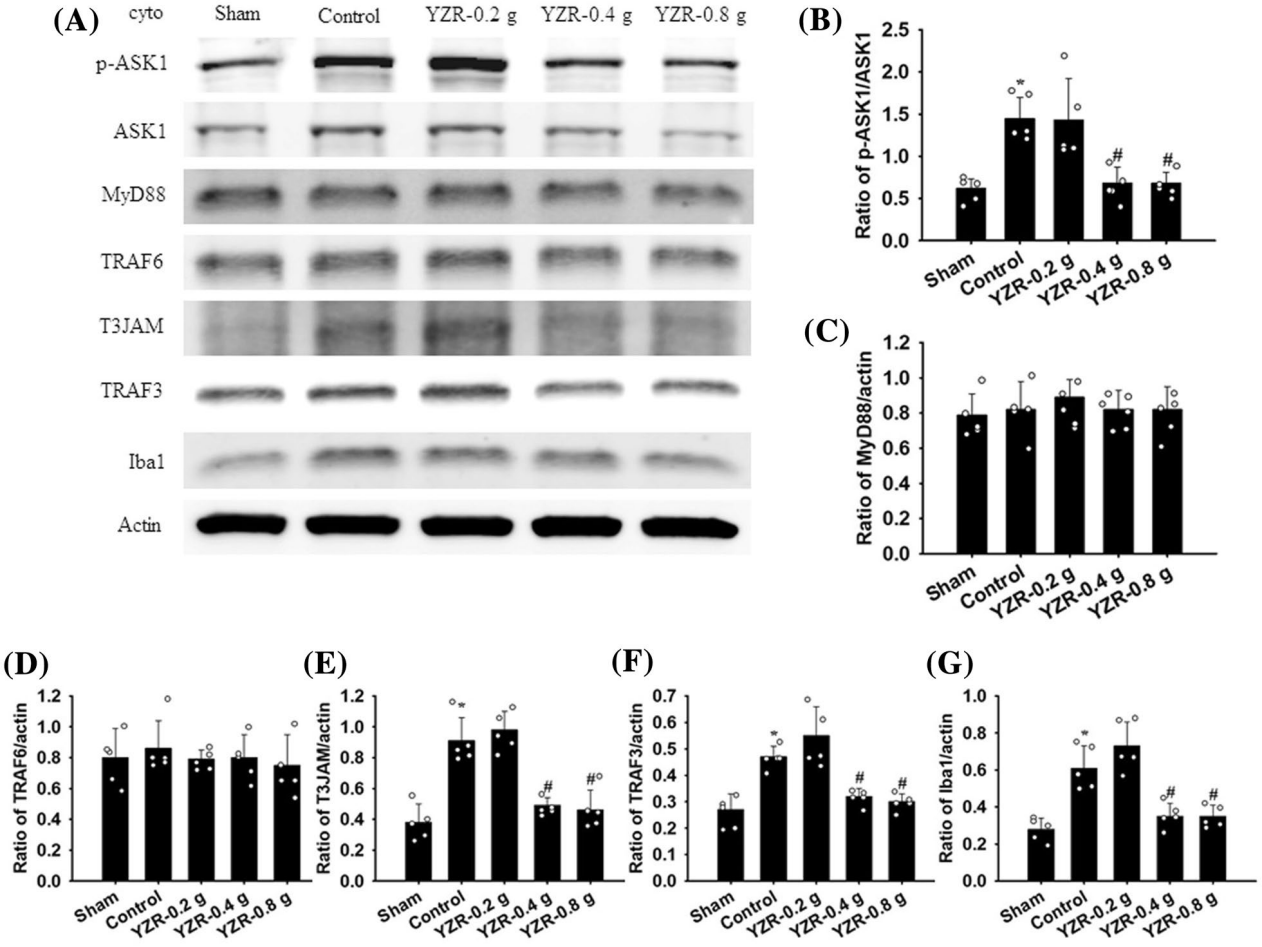
Effects of YZR-0.4 g and YZR-0.8 g treatments on cytosolic p-ASK1, ASK, MyD88, TRAF6, T3JAM, TRAF3, and Iba1 expression in the penumbral cortex. **A** Representative Western blot images showed cytosolic p-ASK1, ASK, MyD88, TRAF6, T3JAM, TRAF3, and Iba1 expression in the penumbral cortex in the Sham, Control, YZR-0.2 g, YZR-0.4 g, and YZR-0.8 g groups (n = 5) at 1 day after reperfusion. Actin was used as an internal control in the Western blot analysis. The ratios of **B** p-ASK1/ASK1, **C** MyD88/actin, **D** TRAF6/actin, **E** T3JAM/actin, **F** TRAF3/actin, and **G** Iba1/actin were measured in the penumbral cortex among the experimental groups. cyto, cytosolic fraction. *P < 0.05 vs. the Sham group; ^#^*P* < 0.05 vs. the Control group

**Fig. 4:**
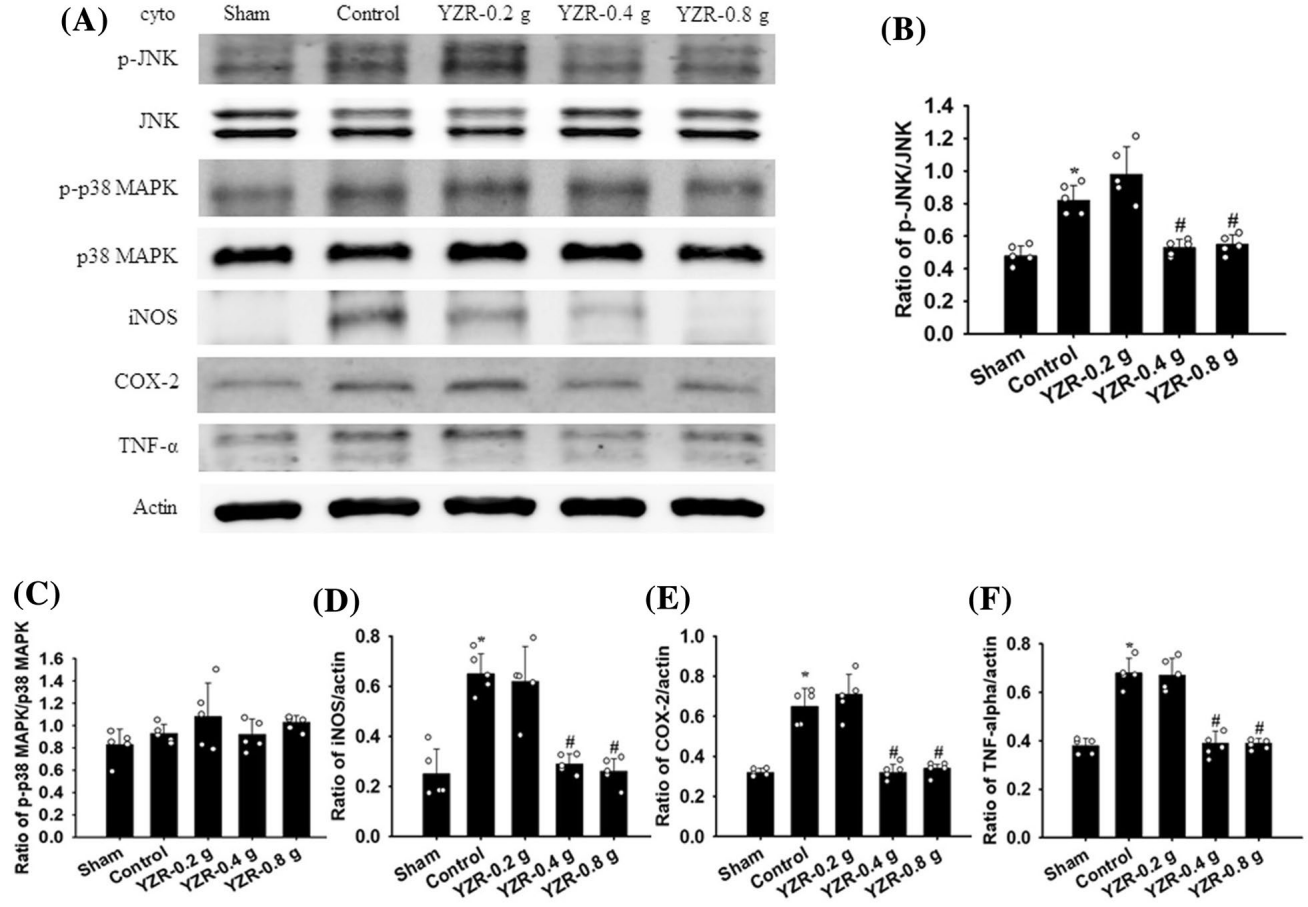
Effects of YZR-0.4 g and YZR-0.8 g treatments on cytosolic p-JNK, JNK, p-p38 MAPK, p38 MAPK, iNOS, COX-2, and TNF-α expression in the penumbral cortex. **A** Representative Western blot images showed cytosolic p-JNK, JNK, p-p38 MAPK, p38 MAPK, iNOS, COX-2, and TNF-α expression in the penumbral cortex in the Sham, Control, YZR-0.2 g, YZR-0.4 g, and YZR-0.8 g groups (n = 5) at 1 day after reperfusion. The ratios of **B** p-JNK/JNK, **C** p-p38 MAPK/p38 MAPK, **D** iNOS/actin, **E** COX-2/actin, and **F** TNF-α/actin were measured in the penumbral cortex among the experimental groups. *P < 0.05 vs. the Sham group; ^#^*P* < 0.05 vs. the Control group

The cytosolic expression of p-ASK1/ASK1, T3JAM/actin, TRAF3/actin, Iba1/actin, p-JNK/JNK, iNOS/actin, COX-2/actin, and TNF-α/actin in the penumbral cortex was markedly higher in the Control group (2.3-, 2.4-, 1.7-, 2.2-, 1.7-, 2.6-, 2.0-, and 1.8-fold, respectively) than in the Sham group (all *P* < 0.05) and was markedly lower in the YZR-0.4 g (0.5-, 0.5-, 0.7-, 0.6-, 0.6-, 0.4-, 0.5-, and 0.6-fold, respectively) and YZR-0.8 g (0.5-, 0.5-, 0.6-, 0.6-, 0.7-, 0.4-, 0.5-, and 0.6-fold, respectively) groups than in the Control group at 1 day after reperfusion (all *P* < 0.05; Figs. 3A, B, E–G, 4A, B, and D–F) [(*F*_4,20_ = 12.359, *P* = 0.000), (*F*_4,20_ = 27.241, *P* = 0.000), (*F*_4,20_ = 19.613, *P* = 0.000), (*F*_4,20_ = 21.942, *P* = 0.000), (*F*_4,20_ = 26.072, *P* = 0.000), (*F*_4,20_ = 26.183, *P* = 0.000), (*F*_4,20_ = 48.012, *P* = 0.000), and (*F*_4,20_ = 52.574, *P* = 0.000), respectively]. However, the cytosolic expression of these proteins did not differ significantly between the Control and YZR-0.2 g groups (*P* > 0.05). In addition, the cytosolic expression of MyD88/actin, TRAF6/actin, and p-p38 MAPK/p38 MAPK in the penumbral cortex did not differ significantly among the experimental groups (*P* > 0.05; Figs. 3A, C, D, 4A and C) [(*F*_4,20_ = 0.456, *P* = 0.767), (*F*_4,20_ = 0.325, *P* = 0.858), and (*F*_4,20_ = 1.788, *P* = 0.171), respectively].

### Effects of D+YZR-0.8 g and SP treatments on cerebral infarction

**Fig. 5:**
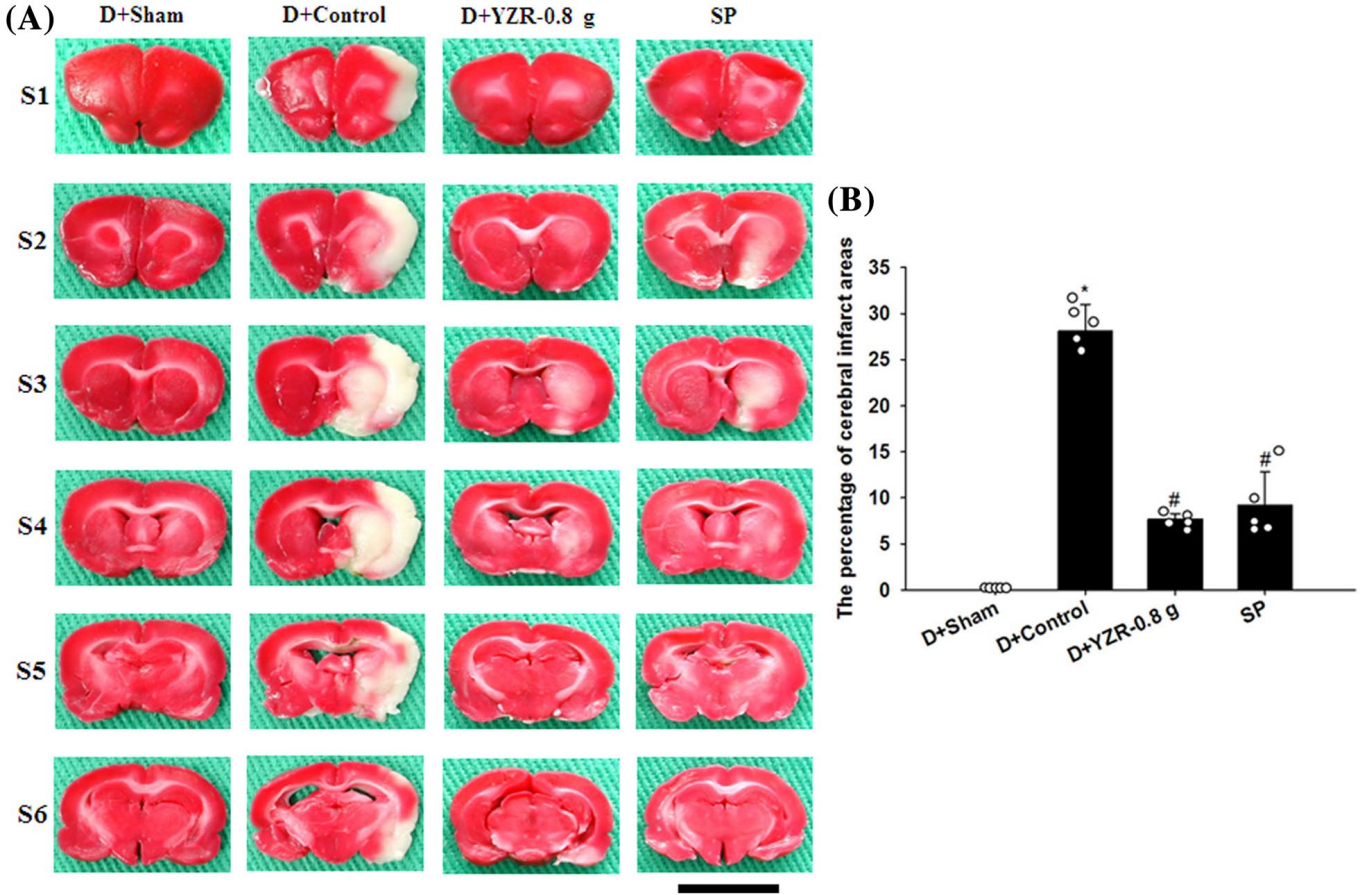
Effects of D+YZR-0.8 g and SP treatments on cerebral infarct areas at 1 day after reperfusion. **A** Representative TTC staining images (S1–S6) in the D+Sham, D+Control, D+YZR-0.8 g, and SP groups (n = 5) showed cerebral infarction. Scale bar represents 1 cm. **B** The percentage of cerebral infarct areas was measured among the experimental groups at 1 day after reperfusion. *P < 0.05 vs. the D+Sham group; ^#^*P* < 0.05 vs. the D+Control group

The percentage of cerebral infarct areas was markedly higher in the D+Control group than in the D+Sham group (*P* < 0.05) and was markedly lower in the D+YZR-0.8 g and SP groups than in the D+Control group at 1 day after reperfusion (both *P* < 0.05; Fig. 5A and B) (*F*_3,16_ = 128.346, *P* = 0.000).

**Fig. 6:**
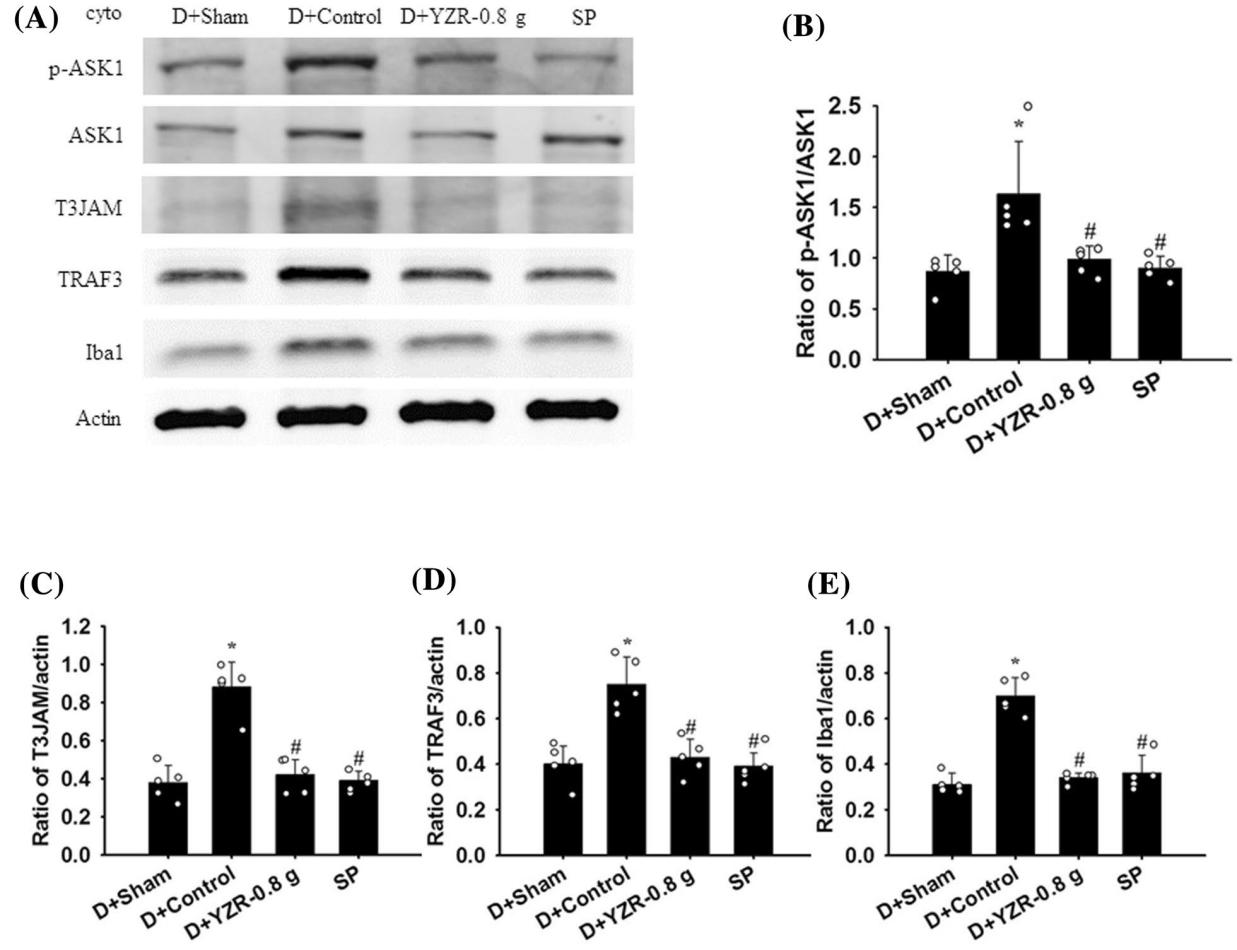
Effects of D+YZR-0.8 g and SP treatments on cytosolic p-ASK1, ASK1, T3JAM, TRAF3, and Iba1 expression in the penumbral cortex. **A** Representative Western blot images showed cytosolic p-ASK1, ASK1, T3JAM, TRAF3, and Iba1 expression in the penumbral cortex in the D+Sham, D+Control, D+YZR-0.8 g, and SP groups (n = 5) at 1 day after reperfusion. The ratios of **B** p-ASK1/ASK1, **C** T3JAM/actin, **D** TRAF3/actin, and **E** Iba1/actin were measured in the penumbral cortex among the experimental groups. *P < 0.05 vs. the D+Sham group; ^#^*P* < 0.05 vs. the D+Control group

**Fig. 7:**
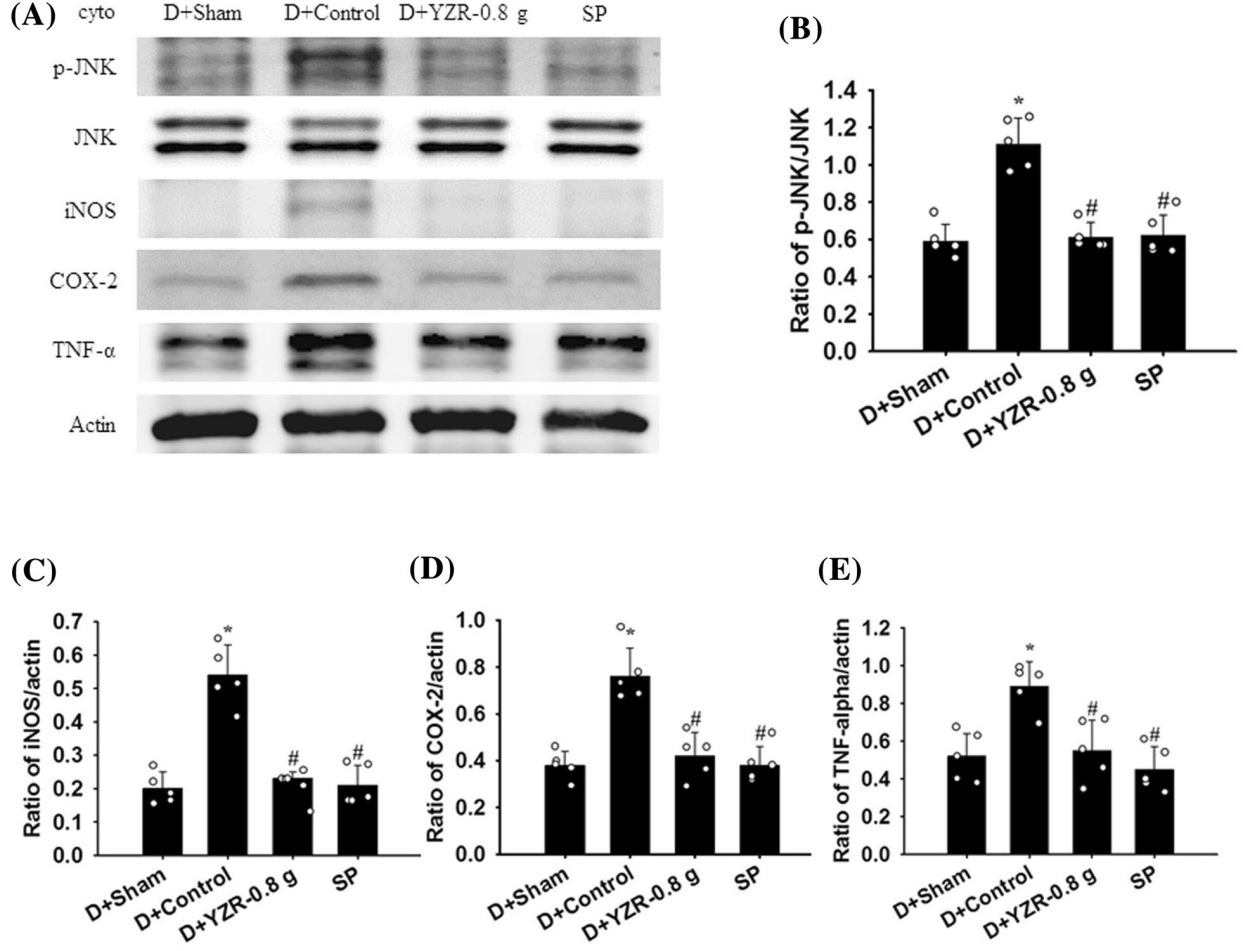
Effects of D+YZR-0.8 g and SP treatments on cytosolic p-JNK, JNK, iNOS, COX-2, and TNF-α expression in the penumbral cortex. **A** Representative Western blot images showed cytosolic p-JNK, JNK, iNOS, COX-2, and TNF-α expression in the penumbral cortex in the D+Sham, D+Control, D+YZR-0.8 g, and SP groups (n = 5) at 1 day after reperfusion. The ratios of **B** p-JNK/JNK, **C** iNOS/actin, **D** COX-2/actin, and **E** TNF-α/actin were measured in the penumbral cortex among the experimental groups. *P < 0.05 vs. the D+Sham group; ^#^*P* < 0.05 vs. the D+Control group

### Effects of D+YZR-0.8 g and SP treatments on neurological function

The NDSs of motor, sensory, and beam balance functions were markedly higher in the D+Control group than in the D+Sham group (all *P* < 0.05) and were markedly lower in the D+YZR-0.8 g and SP groups than in the D+Control groups (all *P* < 0.05; Table 4).

**Table 4:** The NDSs of neurological tests performed in Experiment C and D (n = 10)

Group	NDSs of motor function	NDSs of sensory function	NDSs of beam balance function
D+Sham	0 (0−0)	0 (0−0)	0 (0−0)
D+Control	4 (3−4)*	2 (1−2)*	2 (2−3)*
D+YZR-0.8 g	2 (1−3)#	1 (0−1)^#^	1 (0−1)^#^
SP	3 (2−3)#	1 (0−1)^#^	1 (0−1)^#^

Each value was expressed as median (min−max)

*P < 0.05 vs. the D+Sham group; ^#^*P* < 0.05 vs. the D+Control group

### Effects of D+YZR-0.8 g and SP treatments on the cytosolic expression of p-ASK1, ASK1, T3JAM, TRAF3, Iba1, p-JNK, JNK, iNOS, COX-2, and TNF-α

The cytosolic expression of p-ASK1/ASK1, T3JAM/actin, TRAF3/actin, Iba1/actin, p-JNK/JNK, iNOS/actin, COX-2/actin, and TNF-α/actin in the penumbral cortex was markedly higher in the D+Control group (1.9-, 2.3-, 1.9-, 2.3-, 1.9-, 2.7-, 2.0-, and 1.8-fold, respectively) than in the D+Sham group (all *P* < 0.05) and was markedly lower in the D+YZR-0.8 g (0.6-, 0.5-, 0.6-, 0.5-, 0.5-, 0.4-, 0.6-, and 0.6-fold, respectively) and SP (0.6-, 0.4-, 0.5-, 0.5-, 0.6-, 0.4-, 0.5-, and 0.6-fold, respectively) groups than in the D+Control group at 1 day after reperfusion (all *P* < 0.05; Figs. 6A–E and 7A–E) [(*F*_3,16_ = 7.963, *P* = 0.002), (*F*_3,16_ = 32.593, *P* = 0.000), (*F*_3,16_ = 19.315, *P* = 0.000), (*F*_3,16_ = 42.457, *P* = 0.000), (*F*_3,16_ = 27.482, *P* = 0.000), (*F*_3,16_ = 37.871, *P* = 0.000), (*F*_3,16_ = 20.725, *P* = 0.000), and (*F*_3,16_ = 10.819, *P* = 0.000), respectively].

### Effects of YZR extract and SP treatments on the expression of Iba1, TLR4, GFAP, NF-κB, iNOS, and IL-6

**Fig. 8:**
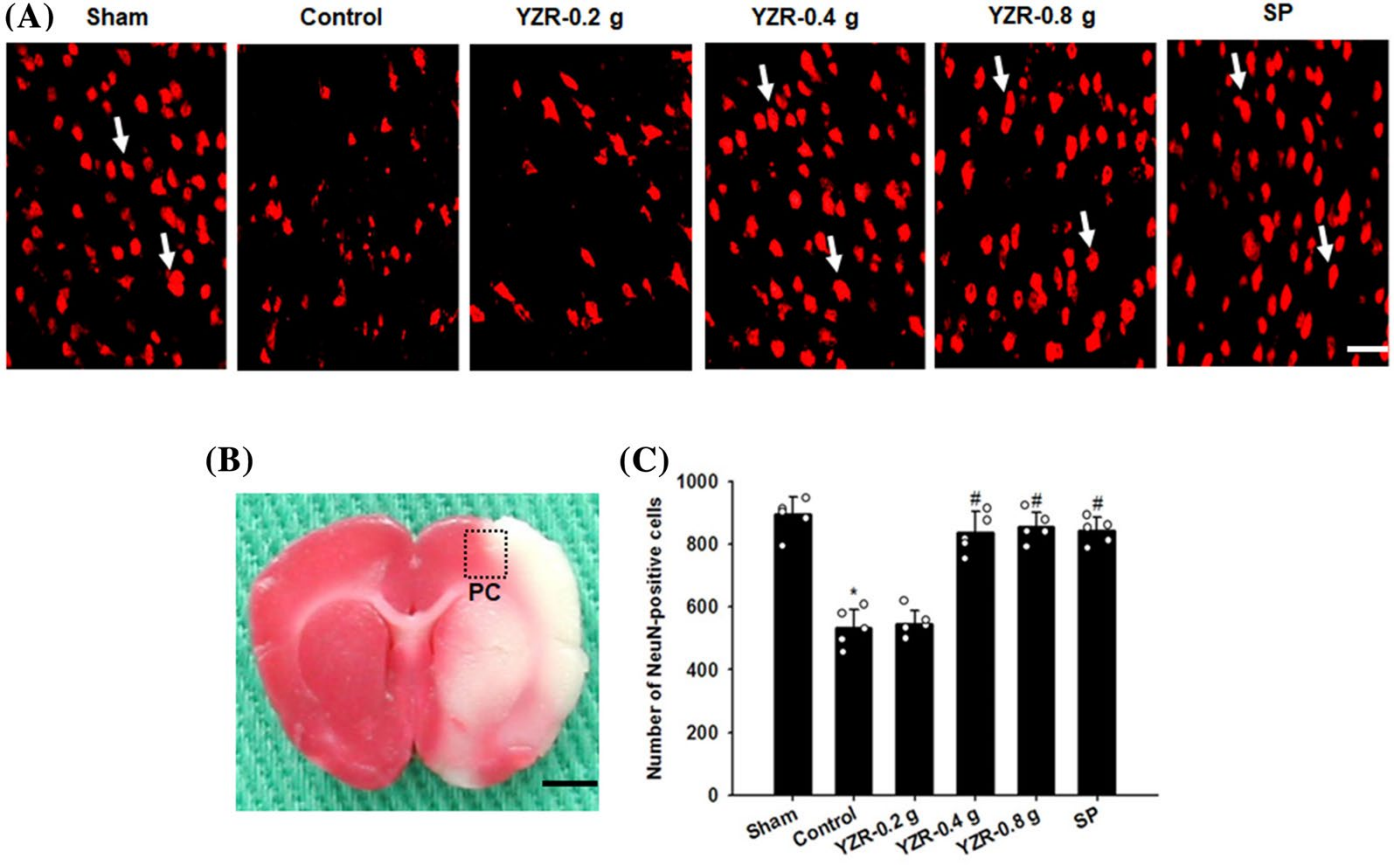
Effects of YZR-0.4 g and YZR-0.8 g treatments on neuronal protection at 1 day after reperfusion. **A** Representative images showed NeuN expression in the penumbral cortex in the Sham, Control, YZR-0.2 g, YZR-0.4 g, YZR-0.8 g, and SP groups (n = 5) at 1 day after reperfusion. **B** A representative TTC staining image showed a coronal brain section. The dashed line square indicates the region where immunopositive cells were counted. PC: penumbral cortex. Dashed line square = 1 mm^2^. **C** The bar graphs showed the number of NeuN-positive cells in the penumbral cortex among the experimental groups. *P < 0.05 vs. the Sham group; ^#^*P* < 0.05 vs. the Control group. Arrows in **A** indicate NeuN-positive cells. Scale bars represent **A** 50 μm and **B** 2 mm

**Fig. 9:**
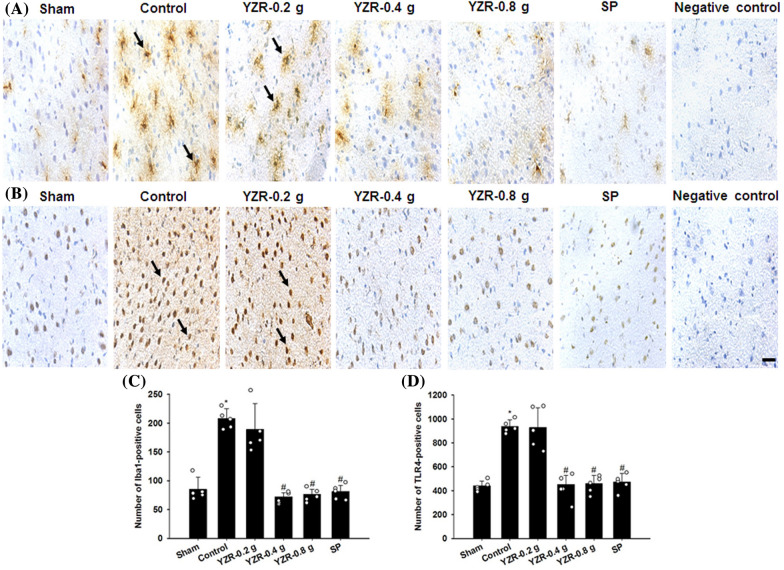
Effects of YZR-0.4 g, YZR-0.8 g, and SP treatments on Iba1 and TLR4 expression in the penumbral cortex. Representative images showed **A** Iba1 and **B** TLR4 expression in the penumbral cortex in the Sham, Control, YZR-0.2 g, YZR-0.4 g, YZR-0.8 g, and SP groups (n = 5) at 1 day after reperfusion. The bar graphs showed the numbers of **C** Iba1- and **D** TLR4-positive cells in the penumbral cortex among the experimental groups. *P < 0.05 vs. the Sham group; ^#^*P* < 0.05 vs. the Control group. Arrows in **A** and **B** indicate Iba1- and TLR4-positive cells, respectively. Scale bar represents 40 μm

**Fig. 10:**
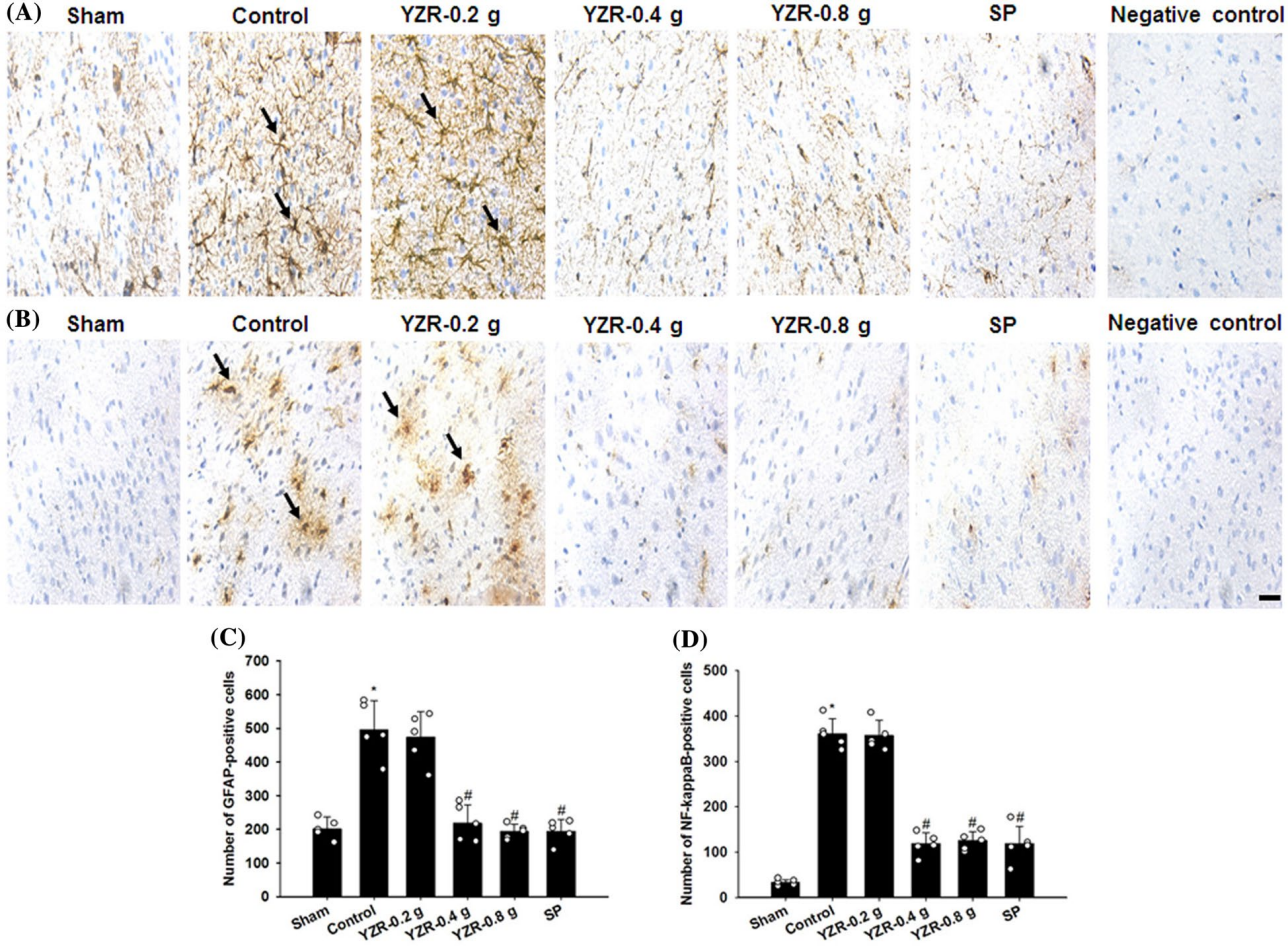
Effects of YZR-0.4 g, YZR-0.8 g, and SP treatments on GFAP and NF-κB expression in the penumbral cortex. Representative images showed **A** GFAP and **B** NF-κB expression in the penumbral cortex in the Sham, Control, YZR-0.2 g, YZR-0.4 g, YZR-0.8 g, and SP groups (n = 5) at 1 day after reperfusion. The bar graphs showed the numbers of **C** GFAP- and **D** NF-κB-positive cells in the penumbral cortex among the experimental groups. *P < 0.05 vs. the Sham group; ^#^*P* < 0.05 vs. the Control group. Arrows in **A** and **B** indicate GFAP- and NF-κB-positive cells, respectively. Scale bar represents 40 μm

**Fig. 11:**
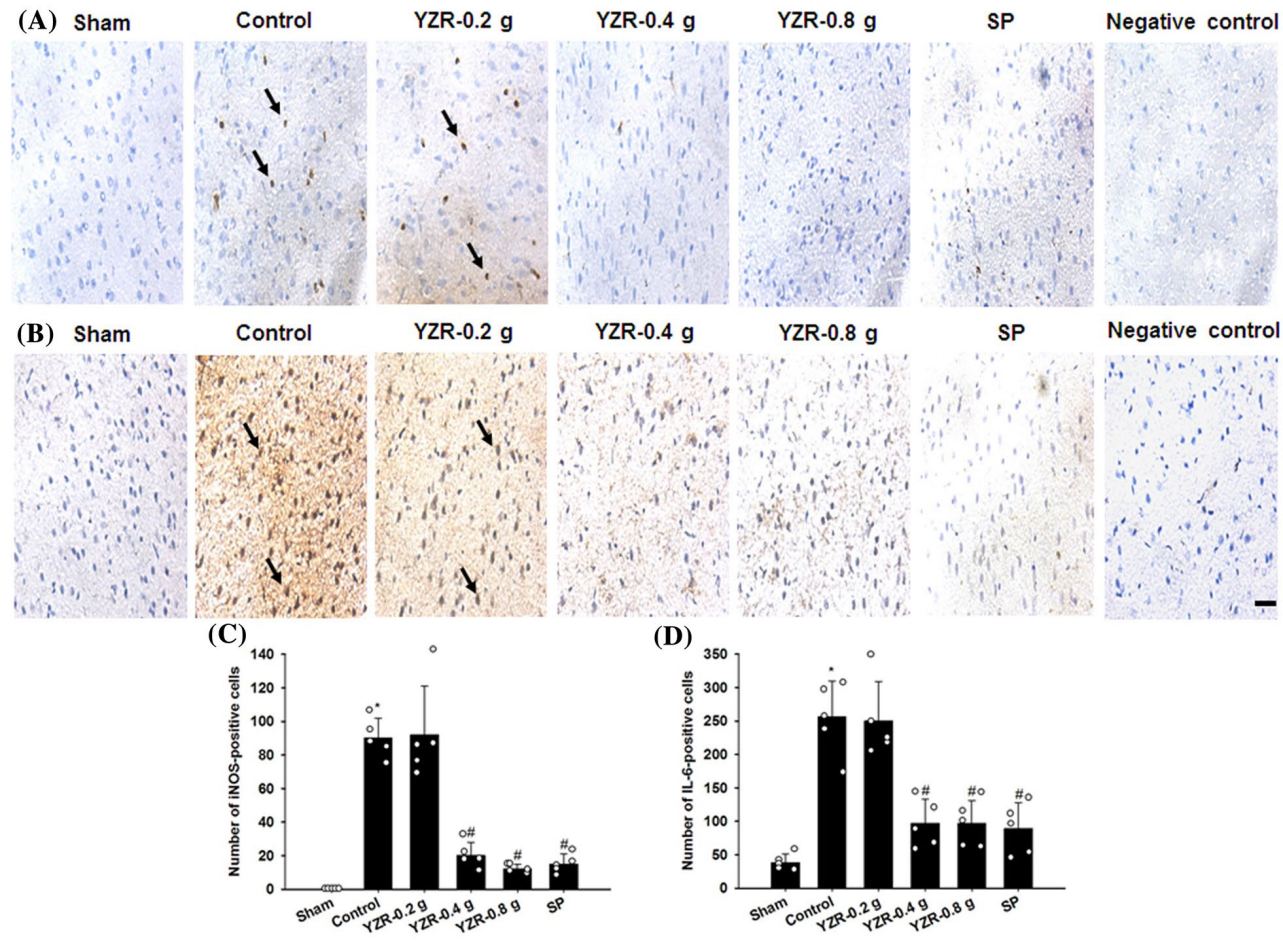
Effects of YZR-0.4 g, YZR-0.8 g, and SP treatments on iNOS and IL-6 expression in the penumbral cortex. Representative images showed **A** iNOS and **B** IL-6 expression in the penumbral cortex in the Sham, Control, YZR-0.2 g, YZR-0.4 g, YZR-0.8 g, and SP groups (n = 5) at 1 day after reperfusion. The bar graphs showed the numbers of **C** iNOS- and **D** IL-6-positive cells in the penumbral cortex among the experimental groups. *P < 0.05 vs. the Sham group; ^#^*P* < 0.05 vs. the Control group. Arrows in **A** and **B** indicate iNOS- and IL-6-positive cells, respectively. Scale bar represents 40 μm

In the present study, the immunopositive cells were detected in the selected penumbral cortex (Fig. 8B). The numbers of Iba1-, TLR4-, GFAP-, NF-κB-, iNOS-, and IL-6-positive cells in the penumbral cortex were markedly higher in the Control group than in the Sham group (all *P* < 0.05) and were markedly lower in the YZR-0.4 g, YZR-0.8 g, and SP groups than in the Control group at 1 day after reperfusion (all *P* < 0.05; Figs. 9A–D, 10A–D, and 11A–D) [(*F*_5,24_ = 38.712, *P* = 0.000), (*F*_5,24_ = 34.085, *P* = 0.000), (*F*_5,24_ = 33.126, *P* = 0.000), (*F*_5,24_ = 126.919, *P* = 0.000), (*F*_5,24_ = 46.320, *P* = 0.000), and (*F*_5,24_ = 24.066, *P* = 0.000), respectively]. However, no significant differences were found in immunopositive cell numbers between the Control and YZR-0.2 g groups (*P* > 0.05).

### Expression of neuronal nuclei-, TLR4/Iba1-, TLR4/GFAP-, and NF-κB/GFAP-positive cells, and activated forms of Iba1 and GFAP in the penumbral cortex

**Fig. 12:**
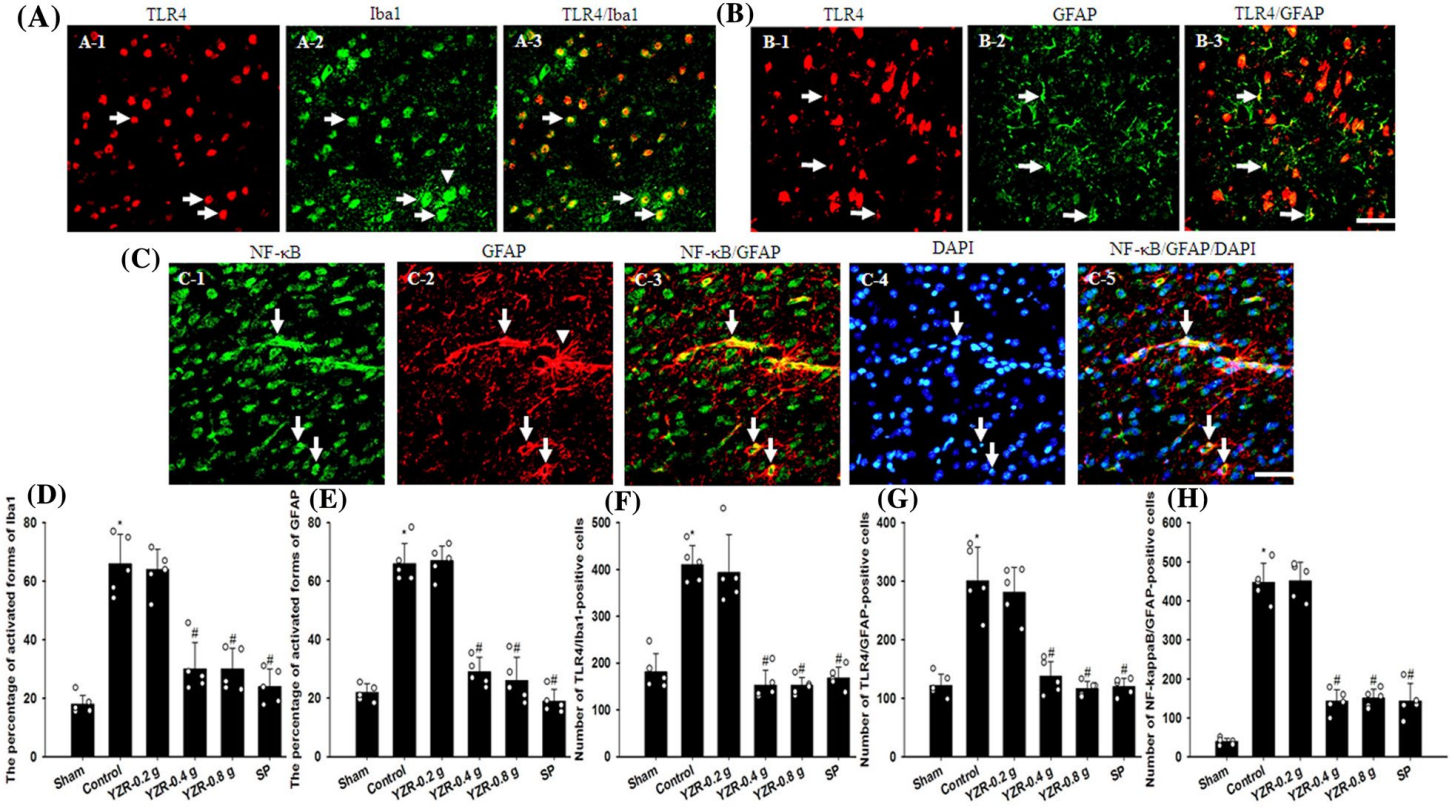
Expression of TLR4/Iba1, TLR4/GFAP, and NF-κB/GFAP, and activated forms of Iba1 and GFAP in the penumbral cortex. Arrows in **A-1**, **A-2**, **B-1**, **B-2**, **C-1**, **C-2**, and **C-4** indicate TLR4-, Iba1-, TLR4-, GFAP-, NF-κB-, GFAP-, and DAPI-positive cells, respectively. Arrows in **A-3**, **B-3**, and **C-3** indicate TLR4/Iba1, TLR4/GFAP, and NF-κB/GFAP double-labeled cells, respectively. Arrows in **C-5** indicate NF-κB/GFAP-positive cells labeled with DAPI. Arrowheads in **A-2** and **C-2** indicate activated forms of Iba1 and GFAP, respectively. The bar graphs showed the percentages of activated forms of **D** Iba1 and **E** GFAP in the penumbral cortex among the experimental groups (n = 5) at 1 day after reperfusion. In addition, the bar graphs showed the numbers of **F** TLR4/Iba1- **G** TLR4/GFAP-, and **H** NF-κB/GFAP-positive cells in the penumbral cortex among the experimental groups (n = 5). *P < 0.05 vs. the Sham group; ^#^*P* < 0.05 vs. the Control group. Scale bars in **B-3** and **C-5** represent 50 μm

TLR4-, Iba1-, GFAP-, and NF-κB-positive cells were predominantly expressed in the selected penumbral cortex (Figs. 12A-1, A-2, B-1, B-2, C-1, and C-2). Iba1- and GFAP-positive cells colocalized with TLR4 (Fig. 12A-3 and B-3). GFAP-positive cells colocalized with NF-κB (Fig. 12C-3). NF-κB double labeling with GFAP was detected in the nucleus (Fig. 12C-4 and C-5). The numbers of TLR4/Iba1-, TLR4/GFAP-, and NF-κB/GFAP-positive cells in the penumbral cortex were markedly higher in the Control group than in the Sham group (all *P* < 0.05) and were markedly lower in the YZR-0.4 g, YZR-0.8 g, and SP groups than in the Control group at 1 day after reperfusion (all *P* < 0.05; 12F–H) [(*F*_5,24_ = 39.640, *P* = 0.000), (*F*_5,24_ = 34.769, *P* = 0.000), and (*F*_5,24_ = 113.297, *P* = 0.000), respectively]. By contrast, the number of neuronal nuclei (NeuN)-positive cells in the penumbral cortex was markedly lower in the Control group than in the Sham group (*P* < 0.05) and was markedly higher in the YZR-0.4 g, YZR-0.8 g, and SP groups than in the Control group at 1 day after reperfusion (*P* < 0.05; Fig. 8A and C) (*F*_5,24_ = 47.439, *P* = 0.000). However, no significant differences were found in immunopositive cell numbers between the Control and YZR-0.2 g groups (*P* > 0.05). The activated forms of Iba1 (activated microglia)- and GFAP (reactive astrocytes)-positive cells are morphologically characterized by swollen processes with amoeboid cell bodies and hypertrophy of main cellular processes, respectively (Fig. 12A-2 and C-2). The percentages of activated forms of Iba1 and GFAP in the penumbral cortex were markedly higher in the Control group than in the Sham group (both *P* < 0.05), and were markedly lower in the YZR-0.4 g, YZR-0.8 g, and SP groups (all *P* < 0.05; Fig. 12D and E) [(*F*_5,24_ = 37.792, *P* = 0.000) and (*F*_5,24_ = 77.384, *P* = 0.000), respectively]. No significant differences were found in the percentages of activated forms of Iba1 and GFAP between the Control and YZR-0.2 g groups (*P* > 0.05).

## Discussion

Post-ischemic inflammation, one of the main pathological features in the early stage of cerebral I/R injury, contributes to the release of pro-inflammatory mediators, which exacerbate cerebral infarction [39]. Studies have reported that TLR4 expressed on microglia and astrocytes plays a crucial role in the generation of pro-inflammatory cytokines in the initial stage of cerebral I/R injury, whereas pharmacological interventions alleviate cerebral infarction by inhibiting TLR4-mediated microglial and astrocytic activation in the ischemic area in the acute phase of transient MCAo [40]. In the present study, the TTC-stained brain sections revealed that cerebral infarction was predominantly distributed in the right cerebral hemisphere involving the cortex and striatum at 1 day after 90 min of MCAo. However, the YZR extract administered at doses of 0.4 g/kg (YZR-0.4 g) and 0.8 g/kg (YZR-0.8 g), but not 0.2 g/kg (YZR-0.2 g), significantly reduced infarct areas and effectively alleviated behavioral deficits (including motor, sensory, and beam balance functions). TTC staining results also revealed that YZR-0.8 g treatment could fully reverse cortical infarction in cerebral I/R injury. In addition, our Western blot, IHC, and IF results revealed that the expression of Iba1 (a marker of microglia), TLR4, and GFAP (a marker of reactive astrocytes) was markedly increased in the penumbral cortex at 1 day after reperfusion. In cerebral ischemia, the transformation of microglia morphology into an amoeboid cell shape in the ischemic area is widely utilized to determine microglia activation [41]. Moreover, activated microglia and reactive astrocytes release inflammatory mediators in the ischemia area, leading to the exacerbation of cerebral infarction in the acute phase after cerebral ischemia [42]. Our results further revealed that amoeboid microglia and reactive astrocytes were predominantly expressed in the penumbral cortex, and activated microglia and reactive astrocytes were double labeled with TLR4, whereas YZR-0.4 g and YZR-0.8 g treatments effectively downregulated the increased expression of TLR4, TLR4/Iba1, and TLR4/GFAP, and inhibited microglial and astrocytic activation in the peri-infarct region. By contrast, the expression of NeuN (neuronal marker) was downregulated in the cortical penumbra, whereas YZR extract treatments effectively rescued cortical neurons in the peri-infarct zone. On the basis of these findings, we infer that YZR-0.4 g and YZR-0.8 g treatments effectively reduced cerebral infarct areas and alleviated neurological deficits at 1 day after reperfusion. Moreover, YZR extract treatments exert neuroprotective effects against cerebral infarction partially through the downregulation of TLR4-mediated inflammatory signaling in the acute phase of transient MCAo.

In the pathology of cerebral I/R injury, TLR4 that is mainly expressed in microglia and astrocytes recognizes DAMPs and subsequently elicits downstream inflammatory signaling cascades through MyD88- and TRIF-dependent pathways [3, 4]. In the MyD88-dependent pathway, TLR4 interacts with MyD88 and subsequently stimulates TRAF6, thereby triggering the activation of the downstream targets JNK, p38 MAPK, and NF-κB [3, 43, 44]. Moreover, JNK and p38 MAPK contribute to post-ischemic inflammation and are considered the upstream kinases of NF-κB [18]. In the TRIF-dependent pathway, TLR4 binds to TRIF by interacting with TRAM, and TLR4/TRIF signaling subsequently activates TRAF3. Furthermore, TRAF3 located on the cell membrane cooperates with T3JAM and then promotes the activation of JNK and TLR4, creating a vicious circle and amplifying TLR4-mediated inflammatory signaling [10, 13–15]. Previous studies have reported that TLR4-mediated signaling causes JNK, p38 MAPK, and NF-κB activation, which induces the production of pro-inflammatory factors, including iNOS, COX-2, TNF-α, and IL-6, in the ischemic area, further exaggerating BBB disruption and cerebral infarction. By contrast, pharmacological reduction of the aforementioned inflammatory mediators and cytokines effectively attenuates cerebral infarction at 1 day after MCAo [3, 8, 40, 45]. The current findings indicated that the expression levels of TRAF3, T3JAM, p-JNK, NF-κB, iNOS, COX-2, TNF-α, and IL-6 were markedly higher in the penumbral cortex. However, YZR-0.4 g and YZR-0.8 g treatments effectively reversed the increased expression of the aforementioned proteins but did not affect the expression of MyD88, TRAF6, and p-p38 MAPK in the penumbral cortex at 1 day after reperfusion. In addition, the IF assay revealed that reactive astrocytes colocalized with NF-κB located in the nucleus in the peri-infarct region, indicating the activation and translocation of NF-κB into the nucleus following cerebral I/R injury. In addition, YZR extract treatments effectively downregulated NF-κB activation in the nuclei of reactive astrocytes. The present results indicate that YZR-0.4 g and YZR-0.8 g treatments exert neuroprotective effects against cerebral I/R injury possibly by downregulating the TLR4-mediated TRAF3/T3JAM/JNK, but not MyD88/TRAF6/JNK (p38 MAPK), signaling pathway in the peri-infarct cortex. Furthermore, the effects of YZR extract treatments on cerebral infarction are partially due to the suppression of JNK/NF-κB-mediated iNOS, COX-2, TNF-α, and IL-6 expression in the penumbral cortex at 1 day after reperfusion.

JNK, a member of the MAPK family, is considered a major stress-responsive kinase and is closely involved in the induction of inflammation and apoptosis in the penumbra region after transient focal cerebral ischemia [19]. In the initial stage of cerebral ischemia, phosphorylated JNK induces NF-κB activation, which causes the production of excessive amounts of pro-inflammatory mediators in the ischemic area, subsequently exacerbating cerebral infarction. Thus, JNK plays a critical role in the regulation of post-ischemic inflammation [46]. In addition to JNK activation by TLR4/MyD88- and TLR4/T3JAM-mediated signaling, JNK is activated by ASK1 signaling in ischemic brain injury [16]. In ASK1/JNK signaling, ASK1 activates the downstream MKK4/MKK7-JNK signaling pathway in response to ischemia-induced oxidative stress after transient cerebral ischemia. Moreover, reactive oxygen species and the pro-inflammatory cytokine TNF-α induce ASK1 phosphorylation at Thr-845, which is required for ASK1 kinase activity [16, 47]. Related studies have reported that phosphorylated ASK1 (Thr-845) activates downstream JNK signaling and activated JNK then translocates into the nucleus and modulates stress-responsive transcription factors (such as NF-κB and activator protein 1), which induce gene transcription, resulting in inflammation and apoptosis in the ischemic area following cerebral I/R injury [17, 46]. In a previous study, increased activation of ASK1/JNK signaling in the penumbra worsened neurological deficits and exaggerated infarct size at 1 day after transient MCAo [48]. By contrast, pharmacological inhibition of ASK1/JNK signaling markedly reduced the cerebral infarct volume in acute perinatal hypoxic-ischemic cerebral injury [49]. Our Western blot findings revealed that the expression levels of p-ASK1/ASK1 and p-JNK/JNK ratios were markedly upregulated in the penumbral cortex, whereas YZR-0.4 g and YZR-0.8 g treatments effectively reduced the p-ASK1/ASK1 and p-JNK/JNK ratios at 1 day after reperfusion. On the basis of these results, we suggest that YZR extract treatments attenuate cerebral ischemic injury partially through the suppression of ASK1/JNK signaling activation in the peri-infarct cortex. Furthermore, the anti-infarct effects of YZR extract treatments may be partially attributed to the downregulation of TLR4/T3JAM/JNK- and ASK1/JNK-mediated inflammatory signaling in the penumbral cortex at 1 day after reperfusion.

**Fig. 13:**
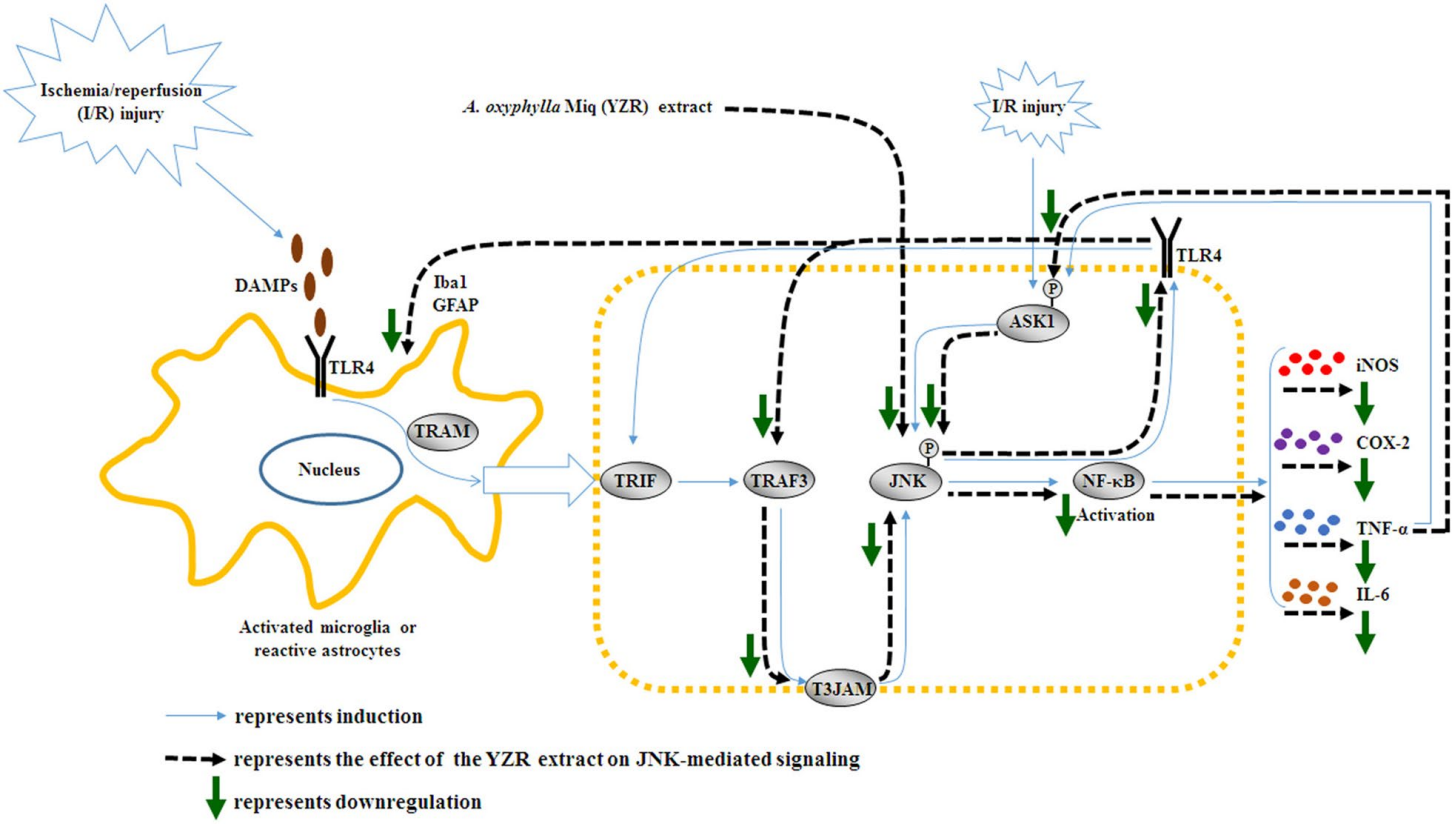
Schematic representation of the possible effects of YZR extract treatments on cerebral infarction induced by the downregulation of the JNK-mediated TLR4/T3JAM- and ASK1-related inflammatory signaling pathways in the penumbral cortex. P: phosphorylated

JNK-mediated inflammatory signaling leads to the production of excessive amounts of the pro-inflammatory mediators, such as TNF-α, which in turn induces ASK1 activation, promoting the activation of ASK1/JNK signaling in cerebral I/R injury [50]. By contrast, pharmacological downregulation of p-JNK attenuated TLR4, Iba1, NF-κB, COX-2, and TNF-α expression in the peri-infarct cortex and striatum; reduced cerebral infarct areas; and ameliorated neurobehavioral deficits in a rat model of permanent MCAo [51]. On the basis of these findings, we propose that activated JNK could in turn regulate its upstream factor expression and play a key role in the regulation of TLR4/JNK- and ASK1/JNK-mediated inflammatory signaling after focal cerebral ischemia. Thus, in the present study, to identify the possible role of JNK in the effects of YZR extract treatments on cerebral infarction after transient MCAo, pretreatment with SP600125, an inhibitor of the JNK pathway, and pretreatment with 1% DMSO were performed in the SP (as the positive control group) and D+YZR-0.8 g groups (as the treatment group), respectively. SP600125, a non-protein synthetic inhibitor of JNK enzymatic activity, inhibits interactions between JNK and its substrates [52]. In the acute phase of cerebral I/R injury, SP600125 treatment reduced cerebral infarction by inhibiting the expression of inflammatory mediators including TNF-α, IL-1β, IL-6, and matrix metalloproteinase-9 and downregulating the mitochondria-mediated apoptotic pathway in the ischemic area [53–55]. In the present study, the Western blotting, IHC, and TTC staining results revealed that SP and D+YZR-0.8 g treatments effectively reversed the increased p-ASK1/ASK1 and p-JNK/JNK ratios, and the increased expression of TLR4, Iba1, GFAP, T3JAM, TRAF3, NF-κB, iNOS, COX-2, TNF-α, and IL-6 in the penumbral cortex and subsequently reduced the percentage of cerebral infarct areas and NDSs at 1 day after reperfusion. In addition, the aforementioned effects of the YZR extract treatment (D+YZR-0.8 g group) on cerebral I/R injury were similar to those of the SP600125 treatment (SP group). According to these findings, we reasonably assume that YZR extract treatments protect against cerebral I/R injury by downregulating JNK-mediated signaling in the penumbral cortex. The inhibitory effect of YZR extract treatments on JNK activation contributes to the suppression of TLR4/Iba1 (GFAP)/TRAF3/T3JAM- and NF-κB/ASK1-mediated signaling, and then abrogates the vicious circles of TLR4/JNK and ASK1/JNK signaling in the penumbral cortex. Thus, YZR extract treatments reduce cerebral infarction partially through the downregulation of JNK-mediated TLR4/T3JAM- and ASK1-related inflammatory signaling at 1 day after reperfusion (Fig. 13).

## Conclusions

The findings of this study indicated that the YZR extract administered at doses of 0.4 g/kg and 0.8 g/kg significantly reduced cerebral infarction and alleviated neurological deficits in the early stage of MCAo; that is, after 90 min of MCAo. Further analysis revealed that YZR extract treatments exert neuroprotective effects against cerebral I/R injury by downregulating the JNK-mediated signaling pathway in the peri-infarct cortex. Furthermore, the anti-infarct effects of YZR extract treatments are partially attributed to the downregulation of the JNK-mediated TLR4/T3JAM- and ASK1-related NF-κB signaling pathways in the penumbral cortex at 1 day after reperfusion. Thus, the results of the present study suggest that the *A. oxyphylla* Miq extract may reduce cerebral infarction in the early phase of cerebral I/R injury. However, to elucidate the precise mechanisms underlying the anti-infarct effects of the YZR extract treatment, further research is needed to clarify the effects of the *A. oxyphylla* Miq extract on the regulation of JNK-mediated apoptotic signaling in the acute phase of transient focal cerebral ischemia.

## Data Availability

The datasets used and/or analysed during the current study are available from the corresponding author upon request.

## Abbreviations

I/R: Ischemia–reperfusion
TLR: Toll-like receptor
DAMP: Damage-associated molecular pattern
MyD88: Myeloid differentiation primary response gene 88
IL: Interleukin
TRIF: Toll/interleukin 1 receptor homology domain-containing adaptor-inducing interferon-β
NF-κB: Nuclear factor-kappa B
SP: SP600125
Iba1: Ionized calcium-binding adapter molecule 1
GFAP: Glial fibrillary acidic protein
TNF: Tumor necrosis factor
TRAF6: TNF receptor-associated factor 6
MAPK: Mitogen-activated protein kinase
JNK: C-Jun N-terminal kinase
TRAM: TRIF-related adaptor molecule
T3JAM: TRAF3-interacting JNK-activating modulator
ASK1: Apoptosis signal-regulating kinase 1
MKK: MAPK kinase
iNOS: Inducible nitric oxide synthase
COX-2: Cyclooxygenase-2
BBB: Blood–brain barrier
MCAo: Middle cerebral artery occlusion
YZR: Yi Zhi Ren
IP: Intraperitoneal
ICV: Intracerebroventricular
NIFDC: National Institutes for Food and Drug Control
RT: Room temperature
HPLC: High-performance liquid chromatography
MCA: Middle cerebral artery
ECA: External carotid artery
ICA: Internal carotid artery
mNSS: Modified neurological severity score
NDS: Neurological deficit score
TTC: 2,3,5-Triphenyltetrazolium chloride
PFA: Paraformaldehyde
NC: Nitrocellulose
DMSO: Dimethyl sulfoxide
PBST: Phosphate buffered saline/Tween 20
IHC: Immunohistochemical
IF: Immunofluorescence
DAPI: 4′,6-Diamidino-2-phenylindole
ANOVA: One-way analysis of variance
NeuN: Neuronal nuclei
AU: Absorbance unit
PC: Penumbral cortex
